# Retraction: T Tubules and Surface Membranes Provide Equally Effective Pathways of Carbonic Anhydrase-Facilitated Lactic Acid Transport in Skeletal Muscle

**DOI:** 10.1371/journal.pone.0295635

**Published:** 2023-12-04

**Authors:** The PLOS ONE Editors

After this article [1] was published, concerns were raised about some of the immunocytochemical images in Fig 1. Specifically:

- Multiple sections of the CA IV panels in Fig 1a in [1] appear similar to sections of Figs 3A-C in [2] but the full panels are not duplicated.
- In Fig 1b, there appear to be similarities between two sets of two areas within the left CA IV panel.

During editorial follow-up on these issues, the authors stated that the histochemical images in Fig 1a in [1] and Figs 3A-C in [2] may have been from serial sections from the same muscle fiber bundle, and that areas in question are similar but not identical. The authors also stated that the raw image data underlying figures of concerns in this article [1] are no longer available.

A member of the *PLOS ONE* Editorial Board reviewed the concerns and authors’ responses, and advised that whilst repetitive transverse lines are expected for this type of experiment due to the striated pattern of transverse tubules, different images or different regions within an image would not be expected to have the level of similarity observed in these figures.

Without the original images we cannot resolve the concerns about the integrity and reliability of results reported in Fig 1. Therefore, the *PLOS ONE* Editors retract this article.

GG, SP, and RJS did not agree with the retraction. JH, AW, WSS, PW, and VE either did not respond directly or could not be reached.
